# A comparative content analysis of newspaper coverage about extreme risk protection order policies in passing and non-passing US states

**DOI:** 10.1186/s12889-022-13374-8

**Published:** 2022-05-16

**Authors:** Amanda J. Aubel, Rocco Pallin, Christopher E. Knoepke, Garen J. Wintemute, Nicole Kravitz-Wirtz

**Affiliations:** 1 Violence Prevention Research Program, Department of Emergency Medicine, University of California Davis School of Medicine, 2315 Stockton Blvd, Sacramento, CA 95817 USA; 2 California Firearm Violence Research Center at UC Davis, 2315 Stockton Blvd, Sacramento, CA 95817 USA; 3 Division of Cardiology, University of Colorado School of Medicine, 13199 East Montview Boulevard, Suite 300, Aurora, CO 80045 USA; 4 Adult and Child Consortium for Outcomes Research and Delivery Science, University of Colorado School of Medicine, 13199 East Montview Boulevard, Suite 300, Aurora, CO 80045 USA

**Keywords:** Firearm policy, Violence prevention, Media framing, Red flag law, Health communication, Extreme risk protection order

## Abstract

**Background:**

Extreme risk protection order (ERPO) laws are a tool for firearm violence prevention (in effect in 19 states), often enacted in the wake of a public mass shooting when media coverage of gun violence tends to spike. We compared news media framing of ERPOs in states that passed and those that considered but did not pass such laws after the 2018 mass shooting in Parkland, Florida.

**Methods:**

We conducted a content analysis of 244 newspaper articles about ERPOs, published in 2018, in three passing (FL, VT, RI) and three non-passing states (PA, OH, CO). Measures included language used, stakeholders mentioned, and scientific evidence cited. We use chi-square tests to compare the proportion of articles with each measure of interest in passing versus non-passing states.

**Results:**

Compared to newspaper coverage of non-passing states, news articles about ERPOs in passing states more often used only official policy names for ERPOs (38% vs. 23%, *p* = .03), used less restrictive language such as “prevent” to describe the process of suspending firearm access (15% vs. 3%, *p* < .01), mentioned gun violence prevention advocacy groups (41% vs. 28%, *p* = .08), and referenced research on ERPOs (17% vs. 7%, *p* = .03). Articles about passing states also more often explicitly stated that a violent event was or could have been prevented by an ERPO (20% vs. 6%, *p* < .01).

**Conclusions:**

Media messaging that frames gun violence as preventable, emphasizes identifiable markers of risk, and draws on data in conjunction with community wisdom may support ERPO policy passage. As more states consider ERPO legislation, especially given endorsement by the Biden-Harris administration, deeper knowledge about successful media framing of these life-saving policies can help shape public understandings and support.

**Supplementary Information:**

The online version contains supplementary material available at 10.1186/s12889-022-13374-8.

## Background

Public mass shootings are a relatively rare form of gun violence, but draw substantial media attention. Research has documented large spikes in news coverage of gun violence and firearm policy immediately following public mass shootings [1, 2]. In this way, mass shootings can function as “focusing events,” opening a window of opportunity through which the news media can amplify and influence public discourse and policymaking on gun violence and its prevention [3–5].

In the aftermath of a mass shooting, the news media exposes the public to competing arguments for and against expanding firearm laws. Research suggests that the strength and volume of these competing arguments, as well as the framing of such issues, can influence public support for and political engagement around specific policy solutions [1]. By deciding which issues to cover (agenda setting) and which aspects of issues to emphasize (framing), the media can influence what is deemed important and in need of a policy response and how an issue’s causes and solutions are understood [1, 3]. This process can have direct impacts on policy by shaping policymakers’ perceptions and indirect effects by shaping public perceptions. In turn, as the policymaking process and its outputs feed back into the perceptions of the public, they can reshape the news media’s agenda [3, 6].

Following the February 2018 mass shooting at Marjory Stoneman Douglas High School in Parkland, Florida, extreme risk protection order (ERPO) laws were chief among the policy proposals put forth to address the gun violence problem. Also known as extreme risk laws or, colloquially, “red flag laws,” ERPOs enable law enforcement officials and, in some states, family or household members, coworkers, and health care providers (“petitioners”) to ask a judge for a civil order to temporarily suspend firearm access for individuals (“respondents”) determined to be at imminent risk for harm to themselves or others, as well as prevent these individuals from purchasing firearms. As commonly reported in the news media [7], many felt the Parkland shooting exemplified the need for ERPO legislation: before the attack, several reports were made to local officials and the FBI regarding the shooter’s concerning behavior and threats, but Florida law enforcement lacked the authority and tools to intervene.

Previous research by our group has documented a dramatic and sustained increase in media coverage of ERPOs after the Parkland shooting, coinciding with widespread consideration of ERPO legislation in states across the country [8]. Prior to the Parkland shooting, only three states had ERPO laws in effect (plus two with similar risk warrant laws); by the end of 2018, eight more states had passed ERPO laws and an additional 16 states had considered them. The extent to which ERPO news coverage differed in states that did and did not pass ERPO laws has not yet been investigated.

To explore the relationship between news media framing of ERPOs and ERPO policy status, we conducted a content analysis of news media about ERPOs following the Parkland shooting and compared coverage about states that passed and did not pass (but considered) ERPO policies in the 2018 legislative session. This study provides insights into how this burgeoning firearm violence prevention strategy is portrayed by the news media, and how such message framing may be related to policy passage. These insights may be used to build support for ERPO laws and to better understand and shape what information the public (including potential petitioners) receives about ERPOs.

## Methods

### News article selection

To examine post-Parkland news coverage of ERPOs while the legislation was under consideration, we identified states where ERPO policy was introduced for the first time after Parkland and considered by the legislature in 2018. This information was ascertained from a legislative tracker maintained by *The Trace* [9]*,* as well as state legislature websites, and yielded the following six states (with the date the legislation was introduced in parentheses): Florida (February 21, 2018), Vermont (February 23, 2018), Rhode Island (February 27, 2018), Pennsylvania (March 5, 2018), Ohio (April 5, 2018), and Colorado (April 30, 2018).

For each state, the study period began the day after Parkland (February 15, 2018) and lasted until either the day of legislation passage or failure in that legislative session (see Figure in Additional file 1). Florida, Rhode Island, and Vermont passed ERPO legislation by the end of 2018 (“passing states”). In the three remaining “non-passing states,” the legislation was postponed indefinitely (Colorado), removed from consideration (Pennsylvania), or failed to pass by the end of that legislative session (Ohio). Although other states considered ERPO legislation in 2018, it was either introduced before our study start date or never received serious deliberation. The average study period was 60 days for passing states and 207 days for non-passing states.

We retrieved news articles from Newsbank and Nexis Uni. We included news articles, editorials, and letters to the editor published in English and in US newspapers, excluding blog posts, press releases, and radio or television transcripts. We conducted independent searches for each of the six states, using the name or abbreviation of that state plus at least one of the following ERPO-related search terms: "risk protection order,” "red flag law,” "gun violence restraining order,” "GVRO,” “firearms restraining order,” "firearms emergency protective order,” “ERPO,” "emergency risk protection order,” "extreme risk protective order,” "extreme risk protection order."

**Fig. 1:**
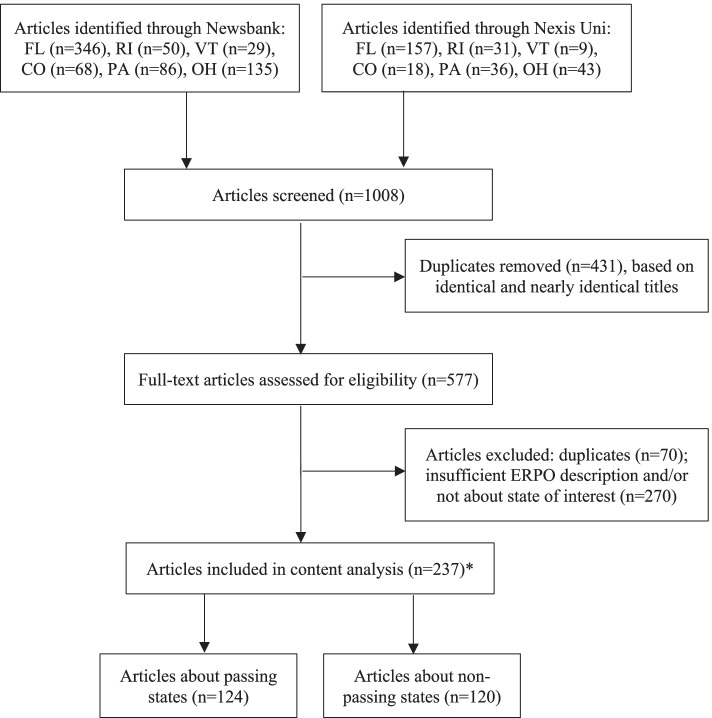
Article search strategy**.** *Of the 237 unique articles, 7 articles were relevant for 2 states and were thus counted twice, creating an analytic sample of 244 articles

Duplicate articles were identified and the most recent (or if equally recent, the longest) version of an article was retained for content analysis. We reviewed full-text articles for relevance, and included only those that (a) contained a description of ERPOs beyond the policy’s name, and (b) discussed ERPO legislation in relation to at least one of our six states. Articles *about* the states being studied rather than simply *published in* those states were included to capture the broader public discourse on ERPOs, which may transcend state boundaries, especially as news is increasingly consumed online. The final analytic sample contained 244 news articles: 124 about ERPOs in passing states and 120 about ERPOs in non-passing states (Fig. 1). A list of these articles and the news outlets in which they were published can be found in Additional file 2; the majority of articles (71%) were published in local news outlets within the state being studied.

### Measures

Guided by a hybrid model of inductive and deductive coding [10], we developed an a priori codebook based on prior media analysis theory and research and added emergent codes, via ongoing discussion among the coding team, to capture additional framing elements in the text. Additional details on our codebook development and coding process have been published elsewhere [8] and are briefly summarized below.

#### Scope of news outlet

Articles published in *The New York Times, The Washington Post, Chicago Tribune, Los Angeles Times, The Wall Street Journal,* and *USA Today* or written by The Associated Press were classified as national in scope; all others were considered local.

#### Language

We identified all policy names used in news articles to refer to specific ERPO legislation or ERPOs in general, and categorized them into those containing the phrase “red flag” versus all other official policy names/acronyms. We also identified the precise language used to describe the ERPO process of suspending respondents’ access to firearms and created categories based on the most commonly used verbs (e.g., seize, remove, take away). In addition, we noted the presence of key terms, such as “due process,” “gun control,” and “warning signs,” when referencing ERPOs.

#### Contextual information

We measured whether articles mentioned specific events (e.g., high-profile mass shootings, other incidents of firearm violence), case details (e.g., perpetrator’s name, victim description, information about firearms used), ERPO laws in other states or at the federal level, and other firearm violence prevention programs or policies (e.g., background checks, bump stock bans). We also measured whether an article explicitly stated that a violent incident was prevented or could have been prevented by using an ERPO.

#### Anecdotal and research evidence

We measured whether articles quoted or mentioned certain stakeholder groups (e.g., politicians/officials, gun violence prevention advocacy groups, firearm industry groups) and cited scientific evidence related to firearm violence generally and on ERPOs specifically. We also measured whether articles stated that ERPOs have been or could be used to prevent suicides, mass shootings, domestic violence, homicides, community violence, or violence among people with mental illness or cognitive impairments, separately.

### Data analysis

We coded all articles that met our inclusion criteria using Dedoose (Version 8.2.14). As described in our prior publication [8], three authors (AJA, RP, NKW) blindly double-coded 20% of the articles that met inclusion criteria and, throughout the process, met biweekly to compare application of the codebook. Instead of computing intercoder reliability, we used an iterative, collaborative approach to assess coding consistency, which also allowed for the formation of emergent codes and themes. Coding discrepancies were discussed and resolved among the coding team and, when necessary, with a fourth author (CEK). After double-coding 20% of articles, we reached consensus and single-coded the remaining articles. All codes were dichotomized in order to calculate the proportion of articles with each item. Descriptive results for individual states can be found in the Table in Additional file 1. We performed Pearson’s chi-square tests in Stata version 15.1 (StataCorp) to compare the proportion of articles with each item in passing versus non-passing states, and controlled for multiple testing using the Benjamini–Hochberg method [11] with a false discovery rate of alpha = 0.05. The adjusted p-values were computed using R version 4.1.2 (R Project for Statistical Computing).

## Results

Of the 244 ERPO-related news articles, 15.2% were national in scope (Table 1). Articles about ERPOs in passing states were significantly more likely to be published in national news outlets than articles about non-passing states (22.6% vs. 7.5%, *p* < 0.01).

**Table 1:** Characteristics of Newspaper Articles About ERPOs in Passing vs. Non-Passing States, 2018^a^

	All Articles (*n* = 244), No. (%)	Passing States^b^ (*n* = 124), No. (%)	Non-Passing States^c^ (*n* = 120), No. (%)	χ^2^ (d.f. = 1)	Adjusted *p*-values^d^
***Scope of news outlet***					
National	37 (15.2)	28 (22.6)	9 (7.5)	10.78	< *.01*
***Language***					
** Name of policy used**					
"Red flag" names only	88 (36.1)	30 (24.2)	58 (48.3)	15.41	< *.01*
Official policy names only	74 (30.3)	47 (37.9)	27 (22.5)	6.85	*.03*
** Removal language used**					
Take away	89 (36.5)	51 (41.1)	38 (31.7)	2.36	.23
Seize	73 (29.9)	30 (24.2)	43 (35.8)	3.94	.09
Seize only	28 (11.5)	4 (3.2)	24 (20.0)	16.89	< *.01*
Remove	70 (28.7)	38 (30.6)	32 (26.7)	0.47	.55
Bar/prohibit/ban/forbid/block	28 (11.5)	21 (16.9)	7 (5.8)	7.40	*.03*
Confiscate	27 (11.1)	16 (12.9)	11 (9.2)	0.87	.45
Prevent	23 (9.4)	19 (15.3)	4 (3.3)	10.27	< *.01*
Prevent only	12 (4.9)	10 (8.1)	2 (1.7)	5.34	*.05*
** Key terms used**					
"gun control"	79 (32.4)	40 (32.3)	39 (32.5)	0.002	.97
"warning signs"; "red flags"	71 (29.1)	45 (36.3)	26 (21.7)	6.32	*.03*
"Second Amendment"	65 (26.6)	25 (20.2)	40 (33.3)	5.41	*.05*
"common sense"; "sensible"	61 (25.0)	19 (15.3)	42 (35.0)	12.59	< *.01*
"due process"	56 (23.0)	26 (21.0)	30 (25.0)	0.56	.53
***Contextual information***					
** Events mentioned**					
Parkland shooting	180 (73.8)	112 (90.3)	68 (56.7)	35.70	< *.01*
Las Vegas shooting	51 (20.9)	22 (17.7)	29 (24.2)	1.52	.35
Sandy Hook shooting	35 (14.3)	21 (16.9)	14 (11.7)	1.38	.37
Other violent incident	85 (34.8)	46 (37.1)	39 (32.5)	0.57	.53
** Case details mentioned**					
Name of perpetrator	64 (26.2)	51 (41.1)	13 (10.8)	28.93	< *.01*
Victim details	35 (14.3)	20 (16.1)	15 (12.5)	0.65	.53
Firearm info	48 (19.7)	34 (27.4)	14 (11.7)	9.58	*.01*
Event was/could have been prevented by an ERPO	32 (13.1)	25 (20.2)	7 (5.8)	10.99	< *.01*
** Program/policy mentioned**					
Any firearm or violence prevention program/policy, excl. ERPOs	106 (43.4)	52 (41.9)	54 (45.0)	0.23	.69
Other states' or federal ERPO	115 (47.1)	67 (54.0)	48 (40.0)	4.82	.07
***Anecdotal and research evidence***					
** Stakeholder quoted or mentioned**					
Official/politician	194 (79.5)	92 (74.2)	102 (85.0)	4.37	.08
Firearm industry group	93 (38.1)	52 (41.9)	41 (34.2)	1.56	.35
Gun violence prevention advocacy group	85 (34.8)	51 (41.1)	34 (28.3)	4.40	.08
** Evidence cited**					
Any evidence related to gun violence	62 (25.4)	35 (28.2)	27 (22.5)	1.05	.42
Evidence on ERPOs	29 (11.9)	21 (16.9)	8 (6.7)	6.14	*.03*
** Uses for ERPOs mentioned**					
Suicide	38 (15.6)	22 (17.7)	16 (13.3)	0.90	.45
Mass shootings	30 (12.3)	15 (12.1)	15 (12.5)	0.01	.97
Mental illness	12 (4.9)	8 (6.5)	4 (3.3)	1.27	.38
Other^e^	12 (4.9)	6 (4.8)	6 (5.0)	0.003	.97

*Notes*

^a^ ERPO = Extreme risk protection order

^b^ Passing states included Florida, Rhode Island, and Vermont

^c^ Non-passing states included Colorado, Pennsylvania, and Ohio

^d^ *p*-values were adjusted for multiple testing using the Benjamini-Hochberg (false discovery rate) method. Significant differences between passing and non-passing states at *p* < .05 are italicized

^e^ Other included domestic violence, homicide, community violence, and violence among people with dementia or cognitive impairments

When describing ERPOs, articles about passing states were more likely to exclusively use official policy names (e.g., ERPO, gun violence restraining order, extreme risk order) (37.9% vs. 22.5%, *p* = 0.03), while articles about non-passing states more often used only names containing the term “red flag” to describe the policy (48.3% vs. 24.2%, *p* < 0.01).

Overall, the verbs “take away” (36.5%), “seize” (29.9%), and “remove” (28.7%) were most commonly used to describe the process of suspending firearm access from ERPO respondents. The less restrictive term “prevent” (as in “prevent access to firearms”) was much less common, appearing in 9.4% of articles overall. Articles about passing states were significantly more likely to use “prevent” (15.3% vs. 3.3%, *p* < 0.01), but also prohibitory language such as “prohibit,” “bar,” “ban,” and “forbid” (16.9% vs. 5.8%, *p* = 0.03). Eight percent of articles about passing states exclusively used “prevent” versus 1.7% of articles on non-passing states (*p* = 0.05). One in five articles (20.0%) about non-passing states exclusively used “seize” versus 3.2% of articles on passing states (*p* < 0.01).

The most commonly used key terms were “gun control” (32.4%), “warning signs” or “red flags” (29.1%), “Second Amendment” (26.6%), “common sense” or “sensible” (25.0%), and “due process” (23.0%). Articles about non-passing states were significantly more likely to use “common sense” or “sensible” (35.0% vs. 15.3%, *p* < 0.01) and “Second Amendment” (33.3% vs. 20.2%, *p* = 0.05), while articles on passing states more frequently used “warning signs” or “red flags” (36.3% vs. 21.7%, *p* = 0.03).

Nearly three-fourths (73.8%) of all articles mentioned the Parkland shooting. Articles about passing states more often mentioned Parkland (90.3% vs. 56.7%, *p* < 0.01) and included the names of perpetrators (41.1% vs. 10.8%, *p* < 0.01) and specific information about the firearms used (27.4% vs. 11.7%, *p* = 0.01). The proportion of articles that described victims of violence (14.3% overall) or that mentioned other mass shootings in Newtown, CT or Las Vegas, NV or any other violent incident did not significantly differ between groups. One in five articles (20.2%) about passing states explicitly stated that a violent event either was or could have been prevented by an ERPO, compared with 5.8% of articles about non-passing states (*p* < 0.01).

Nearly half (43.4%) of all articles mentioned another firearm or violence prevention program or policy; this did not significantly differ between passing and non-passing states. Differences in mentions of ERPO policies (in place or under consideration) in other states or at the federal level between passing and non-passing states (54.0% vs. 40.0%) also did not reach statistical significance after adjustment for multiple testing (*p* = 0.07).

Officials/politicians were the most commonly mentioned stakeholder group, appearing in almost 80% of all articles. Articles about non-passing states more often mentioned at least one official/politician in the discussion of ERPOs (85.0% vs. 74.2%, *p* = 0.08), whereas articles about passing states more frequently mentioned gun violence prevention advocates (41.1% vs. 28.3%, *p* = 0.08); though, these differences did not reach statistical significance after adjustment for multiple testing. Firearm industry groups were mentioned at similar frequencies in coverage of passing and non-passing states (38.1% overall).

Overall, one-quarter (25.4%) of articles cited any type of scientific evidence related to gun violence generally, with no significant difference between passing and non-passing states. However, articles about passing states were significantly more likely to cite evidence on the implementation or effectiveness of ERPOs specifically than articles about non-passing states (16.9% vs. 6.7%, *p* = 0.03).

Fewer than one in six articles explicitly noted that ERPO policies have been or could be used to prevent specific types of firearm violence: 15.6% mentioned suicide, followed by mass shootings (12.3%) and violence among people with mental illness (4.9%). There were no statistically significant differences between passing and non-passing states.

## Discussion

News coverage following the February 2018 mass shooting in Parkland, FL provides a window into the ongoing public discourse about firearm violence and prevention policies, including temporary firearm removal laws. Six states first introduced ERPO policies after Parkland, and three of them passed such laws in the 2018 legislative session (one of the three non-passing states, Colorado, has since passed an ERPO law). Findings from this content analysis highlight several ways that ERPO media coverage appears distinct from coverage of gun violence more generally, as well as elements of coverage that may inform understandings of ERPO policy passage and implementation at the state level.

Past studies suggest that news coverage of gun violence often reinforces the idea that it is an inevitable and intractable problem rather than preventable [2]. Coverage of ERPOs is therefore unique in that it references an inherently solutions-oriented rather than problem-oriented approach to firearm violence. While relatively few articles in our analysis explicitly mentioned that a violent event was or could have been prevented by an ERPO (13.1%), this idea was significantly more likely to be mentioned in articles about passing states than non-passing states.

Evoking such a “prevention frame” in building support for ERPO policy aligns with prior evidence suggesting that the public is attuned to incidents of gun violence in which someone close to the shooter is said to have known something was wrong but lacked the tools to do anything about it [12]. In our analysis, use of the terms “warning signs” or “red flags” in reference to demonstrated signs of concern (but not in policy names) was more common in ERPO coverage about passing than non-passing states.

This focus on identifiable markers of risk for harm is also consistent with expert guidance and higher levels of public support for risk-based (rather than universal) firearm policies and interventions. For example, past research has found widespread public support (> 80%), including among gun owners, for health professionals talking with patients about gun safety in the context of risk reduction, but lower levels of support for such conversations “in general” [13]. A recent study indicates that public support for ERPO policies and personal willingness to use an ERPO across various risk-based scenarios is similarly high (> 70%) [14].

Our results also suggest that policy names may facilitate or hinder public support and political momentum, with coverage about passing states more often using only official ERPO policy names and non-passing states more often using only colloquial “red flag” policy names. The term “red flag law” has been criticized by gun violence prevention experts for being overly vague, stigmatizing individuals with mental illness, and minimizing the level of risk necessary to warrant firearm prohibition [15], whereas the name “extreme risk protection order” has been recommended for widespread use by violence prevention organizations because it “describe[s] the purpose of the law in common language and invoke[s] urgency to reflect the situations wherein the law would be used” [12]. Recent survey data from California also suggest that official policy names and the term “red flag law” are equally recognizable, though public awareness of EPROs is generally low (34%) [14].

Coverage of ERPOs, including articles about passing states, tended to use harsh and prohibitory language, such as “take away,” “seize,” “ban,” and “prohibit,” to describe the process of firearm recovery. Evidence suggests that gun owners may be more likely to support firearm recovery for someone in crisis if language highlights the temporary nature of such action, rather than a permanent prohibition [16]. In our analysis, although most articles used a combination of both prohibitory and preventive language, ERPO articles about passing states were more likely to exclusively use the word “prevent” to describe implementation of the law (e.g., “prevent access to firearms”), whereas articles about non-passing states more often exclusively used the words “seize” or “seizure.” Future research should explore the public’s reactions to variations in recovery language used to describe the ERPO process.

The phrase “gun control” appeared in one-third of articles in our sample. Findings from prior qualitative studies have emphasized the value of culturally-acceptable language, including avoiding “gun control” language, to engage gun owners in suicide prevention strategies that reduce access to firearms [17, 18]. Media analysis of universal background check laws after the 2012 mass shooting in Newtown, CT has also found that “gun control” was mentioned less frequently in news stories published in states that passed such policies compared to news generally [1]. This same study also suggested that framing firearm policies as “common sense” may be an ineffective way to build policy support because it employs rational instead of value-based messaging; similarly, in our study, the terms “common sense” or “sensible” appeared more often in news coverage about states that did not pass ERPO legislation. In contrast, rights-based arguments, which activate the core values associated with gun ownership, may be more powerful than fact-based ones. In our sample, the term “Second Amendment” was used both in support of and in opposition to ERPOs, though it appeared more often in news coverage of non-passing states.

Contrary to recommendations from experts, victim advocates, and news media organizations [19–21], more than one in four articles in our analysis mentioned perpetrators of gun violence by name, particularly the Parkland shooter, and one in five described the specific firearms used. This practice was significantly more common in articles about passing states, though this may in part reflect that Florida—the state in which the Parkland shooting occurred—was included as one of our passing states (see the Table in Additional file 1 for findings by state). Of note, among the six states in our sample, articles about Florida were also most often published in news outlets outside of the state (see Additional file 2). While journalists may be inclined to provide details about perpetrators and their crimes to inform the public or spark action, focusing narrowly on the details of a single event (episodic framing) without looking at the bigger picture can not only obscure preventive, public health-oriented solutions to gun violence, but may also encourage copycat crimes [19].

Consistent with newspaper coverage of other recent public mass shootings, such as the 2015 Umpqua Community College shooting [22], officials/politicians were by far the most commonly mentioned and quoted stakeholders in ERPO coverage overall. While officials/politicians appeared more often in articles about non-passing states, gun violence prevention advocates, such as Everytown for Gun Safety and student advocates, were mentioned more frequently in articles about passing states. This suggests that the public and, in turn, the policymaking process, may benefit from the perspectives of community groups, which may also be more active in states where ERPO legislation was successfully passed.

References to ERPO policies in other states or at the federal level were also more common in passing states than non-passing states. Similarly, although only one in four articles cited scientific evidence related to gun violence generally, articles about passing states were significantly more likely to cite the small but growing body of research about ERPO implementation and effectiveness. These findings point to the value of relevant data, likely in combination with the lived experience and advocacy efforts of those most impacted, for building policy momentum through the media.

### Limitations

Our study has several limitations. First, our results do not imply causation, i.e., whether news media framing led to (or discouraged) policy passage. Policy process scholars have increasingly recognized the relationship between agenda setting in media and politics as a complex system with nonrecursive interactions and multiple feedback loops, rather than a simple linear process [3]. Our findings build on prior evidence suggesting that these processes are integrally related to each other.

Second, these findings characterize print news media about ERPOs after the Parkland shooting in states that had never before considered ERPO policy; as such, they may not be generalizable to news coverage of ERPOs in other states, during different time periods, or on television or radio. In addition, our inclusion criteria (which selected for policy-related articles) resulted in a sample of articles that was more solutions-oriented than news coverage of gun violence in general, but may resemble news coverage following other mass shootings, which research suggests has become increasingly thematic (vs. episodic) over time [22]. The generalizability of our results is strengthened by the geographic, cultural, and political diversity reflected across the six states in our sample.

Third, we operationalized news media framing as the presence or absence of terms, people, events, and other information; in some cases and in future research, further considering the context in which these items appeared may be useful for better understanding the nature and implications of the framing.

## Conclusion

Findings from this content analysis of newspaper articles about ERPOs in passing and non-passing states suggest that the use of official ERPO policy names, messaging that portrays gun violence as preventable through targeted risk reduction, and statements that are grounded in data and community wisdom may be promising strategies for supporting ERPO policy passage. As of March 2022, 19 states and the District of Columbia have enacted ERPO-type laws. Recent endorsement from the Biden-Harris administration, including the development of model ERPO legislation for states [23], suggests that additional states are likely to introduce similar bills in the near-term. If and how such policies are covered in the news may play a role not only in communicating arguments for and against these laws but also in shaping public understanding and building political momentum. The media and policymakers need not wait for another mass tragedy to uplift tools for violence prevention.

## Supplementary Information

**Additional file 1: Figure**. Study Periods for ERPO-Related Newspaper Articles in Six States, 2018. **Table**. Characteristics of Newspaper Articles About ERPOs in Six States, 2018. Displays the findings from the content analysis for each state in our sample. Displays the time periods during which newspaper coverage was retrospectively collected for each state in our sample.

**Additional file 2: Table. **List of Articles, News Outlets, Scope of News Outlet, and Relevant States Included in Content Analysis. Lists the 237 articles included in the content analysis by article headline, name of the news outlet in which the article was published, scope of the news outlet (local or national), and relevant state(s) discussed in the article.

## Data Availability

The data analysed during the current study are available from the Nexis Uni database via Lexis Nexis online (https://www.lexisnexis.com/en-us/professional/academic/nexis-uni.page) and the Newsbank Access World News database (https://www.newsbank.com/libraries/colleges-universities/solutions/top-resources/access-world-news-2022-edition) using the search terms and time periods specified in the Methods section.

## Abbreviations

CO: Colorado
ERPO: Extreme risk protection order
FL: Florida
OH: Ohio
PA: Pennsylvania
RI: Rhode Island
US: United States
VT: Vermont
