# Supplementary material for: A comparative content analysis of newspaper coverage about extreme risk protection order policies in passing and non-passing US states
Source: BMC Public Health. 2022 May 16;22:981. doi: 10.1186/s12889-022-13374-8 (PMC9109361; doi:10.1186/s12889-022-13374-8)
Supplement: Supplementary file 1 — Additional file 1: Figure. Study Periods for ERPO-Related Newspaper Articles in Six States, 2018. Table. Characteristics of Newspaper Articles About ERPOs in Six States, 2018. Displays the findings from the content analysis for each state in our sample. Displays the time periods during which newspaper coverage was retrospectively collected for each state in our sample. [file 12889_2022_13374_MOESM1_ESM.docx]

**Additional File 1**

Aubel AJ, Pallin R, Knoepke CE, Wintemute GJ, Kravitz-Wirtz N. A Comparative Content Analysis of Newspaper Coverage About Extreme Risk Protection Order Policies in Passing and Non-Passing US States.

**Figure.** Study Periods for ERPO-Related Newspaper Articles in Six States, 2018

**Table.** Characteristics of Newspaper Articles About ERPOs in Six States, 2018

**Figure. Study Periods for ERPO-Related Newspaper Articles in Six States, 2018**

|  | Feb | March | | | April | | May | | June | | July | | Aug | | | Sept | | Oct | | Nov | | Dec | |  |  |
| --- | --- | --- | --- | --- | --- | --- | --- | --- | --- | --- | --- | --- | --- | --- | --- | --- | --- | --- | --- | --- | --- | --- | --- | --- | --- |
|  | 15 | 1 | 15 | 29 | 12 | 26 | 10 | 24 | 7 | 21 | 5 | 19 | 2 | 16 | 30 | 13 | 27 | 11 | 25 | 8 | 22 | 6 | 20 |  |  |
|  |  |  |  |  |  |  |  |  |  |  |  |  |  |  |  |  |  |  |  |  |  |  |  |  |  |
| **Passing states** | | |  |  |  |  |  |  |  |  |  |  |  |  |  |  |  |  |  |  |  |  |  |  |  |
| Florida |  |  | Legislation passed 3/8/18 | | | | | |  |  |  |  |  |  |  |  |  |  |  |  |  |  |  |  |  |
| Vermont |  |  |  |  | Legislation passed 4/10/18 | | | | | | |  |  |  |  |  |  |  |  |  |  |  |  |  |  |
| Rhode Island |  |  |  |  |  |  |  |  | Legislation passed 5/31/18 | | | | | | | |  |  |  |  |  |  |  |  |  |
|  |  |  |  |  |  |  |  |  |  |  |  |  |  |  |  |  |  |  |  |  |  |  |  |  |  |
| **Non-passing states** | | |  |  |  |  |  |  |  |  |  |  |  |  |  |  |  |  |  |  |  |  |  |  |  |
| Colorado |  |  |  |  |  |  | Postponed indefinitely by state senate 5/7/18 | | | | | | | | | | | | |  |  |  |  |  |  |
| Pennsylvania |  |  |  |  |  |  |  |  |  |  |  |  |  |  |  |  | Removed from consideration 9/24/18 | | | | | | | | |
| Ohio |  |  |  |  |  |  |  |  |  |  |  |  |  |  |  |  |  |  |  |  |  |  |  |  | |
|  |  |  |  |  |  |  |  |  |  |  |  |  |  |  |  |  | Legislative session ended 12/31/18 | | | | | | | | |

*Notes.* Study periods for all states began the day after the Parkland shooting (2/15/18) and ended when the state's ERPO bill either passed or failed to pass in the 2018 legislative session. The average study period was 60 days for passing states (FL, VT, RI) and 207 days for non-passing states (CO, PA, OH).

**Table. Characteristics of Newspaper Articles About ERPOs in Six States, 2018**

|  | FL  (n=71) | | RI  (n=36) | | VT  (n=17) | | Passing States (n=124) | | CO  (n=13) | | PA  (n=37) | | OH  (n=70) | | Non-Passing States (n=120) | | Total  (n=244) | |
| --- | --- | --- | --- | --- | --- | --- | --- | --- | --- | --- | --- | --- | --- | --- | --- | --- | --- | --- |
|  | No. | % | No. | % | No. | % | No. | % | No. | % | No. | % | No. | % | No. | % | No. | % |
| ***Scope of news outlet*** | | | | | | | | | | | | | | | | | | |
| National | 18 | 25.4 | 8 | 22.2 | 2 | 11.8 | 28 | 22.6 | 0 | 0.0 | 3 | 8.1 | 6 | 8.6 | 9 | 7.5 | 37 | 15.2 |
| ***Language*** | | | | | | | | | | | | | | | | | | |
| **Name of policy used** |  |  |  |  |  |  |  |  |  |  |  |  |  |  |  |  |  |  |
| "Red flag" names only | 15 | 21.1 | 14 | 38.9 | 1 | 5.9 | 30 | 24.2 | 4 | 30.8 | 9 | 24.3 | 45 | 64.3 | 58 | 48.3 | 88 | 36.1 |
| Official policy names only | 35 | 49.3 | 2 | 5.6 | 10 | 58.8 | 47 | 37.9 | 4 | 30.8 | 19 | 51.4 | 4 | 5.7 | 27 | 22.5 | 74 | 30.3 |
| **Removal language used** |  |  |  |  |  |  |  |  |  |  |  |  |  |  |  |  |  |  |
| Take away | 33 | 46.5 | 13 | 36.1 | 5 | 29.4 | 51 | 41.1 | 5 | 38.5 | 9 | 24.3 | 24 | 34.3 | 38 | 31.7 | 89 | 36.5 |
| Seize | 18 | 25.4 | 7 | 19.4 | 5 | 29.4 | 30 | 24.2 | 4 | 30.8 | 15 | 40.5 | 24 | 34.3 | 43 | 35.8 | 73 | 29.9 |
| Seize only | 2 | 2.8 | 1 | 2.8 | 1 | 5.9 | 4 | 3.2 | 2 | 15.4 | 9 | 24.3 | 13 | 18.6 | 24 | 20.0 | 28 | 11.5 |
| Remove | 20 | 28.2 | 11 | 30.6 | 7 | 41.2 | 38 | 30.6 | 6 | 46.2 | 7 | 18.9 | 19 | 27.1 | 32 | 26.7 | 70 | 28.7 |
| Bar/prohibit/ban/forbid/  block | 9 | 12.7 | 9 | 25.0 | 3 | 17.6 | 21 | 16.9 | 1 | 7.7 | 3 | 8.1 | 3 | 4.3 | 7 | 5.8 | 28 | 11.5 |
| Confiscate | 8 | 11.3 | 2 | 5.6 | 6 | 35.3 | 16 | 12.9 | 1 | 7.7 | 5 | 13.5 | 5 | 7.1 | 11 | 9.2 | 27 | 11.1 |
| Prevent | 14 | 19.7 | 5 | 13.9 | 0 | 0.0 | 19 | 15.3 | 0 | 0.0 | 2 | 5.4 | 2 | 2.9 | 4 | 3.3 | 23 | 9.4 |
| Prevent only | 8 | 11.3 | 2 | 5.6 | 0 | 0.0 | 10 | 8.1 | 0 | 0.0 | 2 | 5.4 | 0 | 0.0 | 2 | 1.7 | 12 | 4.9 |
| **Key terms used** |  |  |  |  |  |  |  |  |  |  |  |  |  |  |  |  |  |  |
| "gun control" | 24 | 33.8 | 9 | 25.0 | 7 | 41.2 | 40 | 32.3 | 6 | 46.2 | 11 | 29.7 | 22 | 31.4 | 39 | 32.5 | 79 | 32.4 |
| "warnings signs"; "red flags" | 24 | 33.8 | 17 | 47.2 | 4 | 23.5 | 45 | 36.3 | 0 | 0.0 | 11 | 29.7 | 15 | 21.4 | 26 | 21.7 | 71 | 29.1 |
| "Second Amendment" | 14 | 19.7 | 8 | 22.2 | 3 | 17.6 | 25 | 20.2 | 7 | 53.8 | 10 | 27.0 | 23 | 32.9 | 40 | 33.3 | 65 | 26.6 |
| "common sense"; "sensible" | 7 | 9.9 | 10 | 27.8 | 2 | 11.8 | 19 | 15.3 | 4 | 30.8 | 8 | 21.6 | 30 | 42.9 | 42 | 35.0 | 61 | 25.0 |
| "due process" | 12 | 16.9 | 10 | 27.8 | 4 | 23.5 | 26 | 21.0 | 4 | 30.8 | 7 | 18.9 | 19 | 27.1 | 30 | 25.0 | 56 | 23.0 |
| ***Contextual information*** | | | | | | | | | | | | | | | | | | |
| **Events mentioned** |  |  |  |  |  |  |  |  |  |  |  |  |  |  |  |  |  |  |
| Parkland shooting | 68 | 95.8 | 30 | 83.3 | 14 | 82.4 | 112 | 90.3 | 7 | 53.8 | 26 | 70.3 | 35 | 50.0 | 68 | 56.7 | 180 | 73.8 |
| Las Vegas shooting | 12 | 16.9 | 9 | 25.0 | 1 | 5.9 | 22 | 17.7 | 3 | 23.1 | 9 | 24.3 | 17 | 24.3 | 29 | 24.2 | 51 | 20.9 |
| Newtown shooting | 16 | 22.5 | 4 | 11.1 | 1 | 5.9 | 21 | 16.9 | 1 | 7.7 | 8 | 21.6 | 5 | 7.1 | 14 | 11.7 | 35 | 14.3 |
| Other violent incident | 27 | 38.0 | 8 | 22.2 | 11 | 64.7 | 46 | 37.1 | 7 | 53.8 | 15 | 40.5 | 17 | 24.3 | 39 | 32.5 | 85 | 34.8 |
| **Case details mentioned** |  |  |  |  |  |  |  |  |  |  |  |  |  |  |  |  |  |  |
| Name of perpetrator | 38 | 53.5 | 9 | 25.0 | 4 | 23.5 | 51 | 41.1 | 1 | 7.7 | 5 | 13.5 | 7 | 10.0 | 13 | 10.8 | 64 | 26.2 |
| Victim details | 15 | 21.1 | 3 | 8.3 | 2 | 11.8 | 20 | 16.1 | 3 | 23.1 | 2 | 5.4 | 10 | 14.3 | 15 | 12.5 | 35 | 14.3 |
| Firearm info | 29 | 40.8 | 4 | 11.1 | 1 | 5.9 | 34 | 27.4 | 0 | 0.0 | 8 | 21.6 | 6 | 8.6 | 14 | 11.7 | 48 | 19.7 |
| Event was/could have been prevented by an ERPO | 20 | 28.2 | 5 | 13.9 | 0 | 0.0 | 25 | 20.2 | 1 | 7.7 | 1 | 2.7 | 5 | 7.1 | 7 | 5.8 | 32 | 13.1 |
| **Program/policy mentioned** | |  |  |  |  |  |  |  |  |  |  |  |  |  |  |  |  |  |
| Any firearm or violence prevention program/ policy, excl. ERPOs | 29 | 40.8 | 14 | 38.9 | 9 | 52.9 | 52 | 41.9 | 3 | 23.1 | 20 | 54.1 | 31 | 44.3 | 54 | 45.0 | 106 | 43.4 |
| Other states' or federal ERPO | 41 | 57.7 | 19 | 52.8 | 7 | 41.2 | 67 | 54.0 | 7 | 53.8 | 15 | 40.5 | 26 | 37.1 | 48 | 40.0 | 115 | 47.1 |
| ***Anecdotal and research evidence*** | | | | | | | | | | | | | | | | | | |
| **Stakeholder mentioned** |  |  |  |  |  |  |  |  |  |  |  |  |  |  |  |  |  |  |
| Official/politician | 49 | 69.0 | 31 | 86.1 | 12 | 70.6 | 92 | 74.2 | 9 | 69.2 | 27 | 73.0 | 66 | 94.3 | 102 | 85.0 | 194 | 79.5 |
| Firearm industry group | 23 | 32.4 | 19 | 52.8 | 10 | 58.8 | 52 | 41.9 | 4 | 30.8 | 12 | 32.4 | 25 | 35.7 | 41 | 34.2 | 93 | 38.1 |
| Gun violence prevention advocacy group | 31 | 43.7 | 14 | 38.9 | 6 | 35.3 | 51 | 41.1 | 4 | 30.8 | 13 | 35.1 | 17 | 24.3 | 34 | 28.3 | 85 | 34.8 |
| **Evidence cited** |  |  |  |  |  |  |  |  |  |  |  |  |  |  |  |  |  |  |
| Any evidence related to gun violence | 19 | 26.8 | 10 | 27.8 | 6 | 35.3 | 35 | 28.2 | 3 | 23.1 | 12 | 32.4 | 12 | 17.1 | 27 | 22.5 | 62 | 25.4 |
| Evidence on ERPOs | 14 | 19.7 | 7 | 19.4 | 0 | 0.0 | 21 | 16.9 | 1 | 7.7 | 3 | 8.1 | 4 | 5.7 | 8 | 6.7 | 29 | 11.9 |
| **Uses for ERPOs mentioned** | |  |  |  |  |  |  |  |  |  |  |  |  |  |  |  |  |  |
| Suicide | 12 | 16.9 | 5 | 13.9 | 5 | 29.4 | 22 | 17.7 | 1 | 7.7 | 6 | 16.2 | 9 | 12.9 | 16 | 13.3 | 38 | 15.6 |
| Mass shootings | 8 | 11.3 | 4 | 11.1 | 3 | 17.6 | 15 | 12.1 | 1 | 7.7 | 7 | 18.9 | 7 | 10.0 | 15 | 12.5 | 30 | 12.3 |
| Mental illness | 5 | 7.0 | 3 | 8.3 | 0 | 0.0 | 8 | 6.5 | 0 | 0.0 | 0 | 0.0 | 4 | 5.7 | 4 | 3.3 | 12 | 4.9 |
| Other† | 4 | 5.6 | 1 | 2.8 | 1 | 5.9 | 6 | 4.8 | 1 | 7.7 | 2 | 5.4 | 3 | 4.3 | 6 | 5.0 | 12 | 4.9 |
| *Notes.* ERPO = Extreme risk protection order  †Other included domestic violence, homicide, community violence, and violence among people with dementia or cognitive impairments | | | | | | | | | | | | | | | | | | |
